# Evaluation of cup placement accuracy in computer assisted total hip arthroplasty

**DOI:** 10.1007/s00402-025-05797-w

**Published:** 2025-03-14

**Authors:** Hiroki Kaneta, Takeshi Shoji, Shinichi Ueki, Hiroyuki Morita, Yosuke Kozuma, Nobuo Adachi

**Affiliations:** 1 Department of Orthopaedic Surgery, Graduate School of Biomedical and Health Sciences, Hiroshima University, Hiroshima, Japan; 2 Department of Artificial Joints and Biomaterials, Graduate School of Biomedical and Health Sciences, Hiroshima University, Hiroshima, Japan

**Keywords:** Total hip arthroplasty, Computer-assisted surgery, Robotic system, CT navigation, Cup placement accuracy, Patient-reported outcome measures

## Abstract

**Background:**

Total hip arthroplasty (THA) accuracy has improved significantly with various advances in computer-assisted equipment (CAE), including robotic systems, computed tomography (CT) navigation, and portable navigation. However, no studies have directly compared the accuracy of acetabular cup placement and its impact on patient-reported outcome measures (PROMs) across these three CAE systems. In this study, we aimed to evaluate cup placement accuracy and PROMs in THA using different CAE systems.

**Methods:**

This retrospective analysis included 196 patients (202 hip joints) who underwent THA with three CAE systems from May 2021 to August 2023. Patients were categorized into the robotic system (73 hips), CT navigation (83 hips), and portable navigation (46 hips). Postoperative CT scans measured cup placement angles—radiographic inclination (RI) and radiographic anteversion (RA) —and compared them with preoperative target angles. Anterior-posterior (AP) cup position differences were evaluated by measuring the distance between the acetabular and cup center in the axial view of the postoperative CT scans. PROMs were evaluated using the Japanese Orthopaedic Association Hip Disease Evaluation Questionnaire (JHEQ) at 3 and 12 months.

**Results:**

Demographic characteristics, including age, sex, primary disease, and Body Mass Index, were similar across groups. The robotic system exhibited significantly smaller deviations in ΔRI and ΔRA compared to CT navigation and portable navigation. AP cup position differences were also smaller in the robotic system versus portable navigation; however, the difference between the robotic and CT navigation systems was not statistically significant. Despite the superior precision of cup placement in the robotic system, no significant differences in JHEQ scores were observed among the groups at 3 and 12 months.

**Conclusion:**

Robotic systems demonstrated superior accuracy in cup placement. However, short-term PROMs did not significantly differ, suggesting that PROMs may not solely depend on accurate cup placement. Future research should investigate additional factors influencing PROMs.

## Introduction

Total hip arthroplasty (THA) is an effective surgical procedure that alleviates pain and restores function in patients with hip disorders such as osteoarthritis (OA), osteonecrosis of the femoral head (ONFH), and rheumatoid arthritis [1]. However, improper acetabular cup positioning during THA can result in complications, including dislocation, impingement, prosthetic wear, and revision surgery [2–4], underscoring the importance of accurate placement for long-term success [5, 6].

Recently, computer-assisted equipment (CAE), including robotic-assisted systems, computed tomography (CT)-based navigation, and portable navigation, has gained prominence in enhancing surgical accuracy in THA by providing real-time intraoperative guidance [7–14]. CT-based navigation systems enable precise cup placement using preoperative three-dimensional CT imaging [15]. While highly accurate, this method is more costly and time-consuming than imageless navigation, which does not rely on preoperative CT images but has limited accuracy [15, 16]. Robotic systems, such as the Mako system (Mako; Stryker, Kalamazoo, MI, USA), enhance acetabular reaming and component placement with high accuracy by integrating CT-based navigation with robotic arm control [17–19]. In the United States, robotic-assisted THA is projected to rise from 2.0% in 2018 to 65.8% by 2030 [20]. A new portable navigation system, the augmented reality-based navigation system (AR Hip; Zimmer Biomet Japan, Tokyo, Japan), was released. AR Hip is a portable navigation tool that allows the surgeon to view an acetabular cup image superimposed on the real surgical field via a smartphone [21]. The navigation system automatically determines the gravitational vector with the smartphone’s gyro sensor [22].

Numerous studies evaluate individual CAE systems; however, direct comparisons of their effects on acetabular cup placement accuracy and postoperative patient-reported outcome measures (PROMs) under standardized conditions, including identical postoperative rehabilitation protocols, are lacking. To our knowledge, no studies have systematically compared all three approaches, and thus, no consensus has been established.

In this study, we aimed to evaluate the accuracy of acetabular cup placement and its relationship with postoperative PROMs by comparing three CAE systems—robotic-assisted, CT-based, and portable navigation—in THA. The study’s novelty lies in its comprehensive comparison of these systems under consistent rehabilitation conditions, which has not been thoroughly investigated in previous reports. By focusing on both surgical precision and PROMs, our study provides valuable insights into the effectiveness of CAE systems in THA and may contribute to the development of improved surgical techniques and patient care.

## Materials and methods

### Study participants

This retrospective single-institution study adhered to the Declaration of Helsinki and was approved by the Institutional Review Board of our hospital. Informed consent was obtained from all participants.

The study included 196 patients (202 hip joints) who underwent primary THA using one of three CAE systems from May 2021 to August 2023, with at least 12 months of follow-up. Exclusion criteria were bilateral or revision THA, neurological, knee, or spinal diseases, contralateral hip severe OA, or arthroplasty, nonelective or oncologic THA, and lack of recorded responses to a PROM [23–25]. In particular, spinal disease was defined as any case with lumbar canal stenosis, scoliosis, or vertebral compression fractures identified by whole-spine X-ray evaluation, as these may influence PROMs and spino-pelvic mobility. Patients were categorized into three groups based on the CAE system used: robotic-assisted system (Mako group), CT navigation (CT-navi group), and portable navigation (AR-navi group). All patients participated in our institutional-wide comprehensive Total Hip Arthroplasty pathway program, including uniform perioperative care, a standard institutional postoperative rehabilitation, and pain management protocol.

### Preoperative planning

In all cases, target cup inclination and anteversion angles were determined preoperatively using a CT-based template, with the supine functional pelvic plane (FPP) as the radiographic reference [26]. To enhance the reproducibility of the FPP during CT scanning, a pillow was placed under the sacrum to align the pelvis in the supine position with bilateral anterior iliac spines in the same horizontal plane, using the inter-teardrop line as the mediolateral axis. The target inclination was set at 40°, and the anteversion angle was determined according to Widmer’s combined anteversion theory, adjusting to 15° ± 5° based on individual patient anatomy [27]. In preoperative planning, impingement and bone coverage were carefully assessed using dedicated software, and the cup placement plan was adjusted accordingly to optimize implant positioning and stability. After cup implantation, combined anteversion was fine-tuned by adjusting the stem anteversion to achieve optimal alignment. Preoperative CT scans were obtained with a slice thickness of 1.25 mm for navigation systems and 0.625 mm for robotic-assisted systems to facilitate accurate planning and execution.

### Surgical procedure

THA was performed by a single surgeon using the anterolateral supine approach across all cases. Various CAE systems, including robotic-assisted, CT-based (CT-based hip navigation version 1.1, Stryker Navigation, Freiburg, Germany), and portable navigation, were used to aid in acetabular cup placement. To minimize selection bias, the type of CAE system used for each patient was randomly assigned. A cementless Taperloc microplasty stem with a G7 acetabular cup (Zimmer Biomet Inc., Warsaw, IN) or an Accolade II femoral hip stem with a Trident II Tritanium cup (Stryker, Kalamazoo, MI, USA) was used, paired with highly cross-linked polyethylene and a 32- or 36-mm delta-ceramic head. A cluster-type cup with three holes was employed.

### Robotic-assisted THA

The MAKO system (Stryker) was utilized, incorporating CT-based planning for accurate acetabular reaming and cup placement. Three 4.0 mm tracker pins were placed in the contralateral iliac crest, and surface mapping was performed to match the patient’s acetabulum to the preoperative CT scan. Single-step reaming was guided by robotic arm assistance with the same size as the preoperative plan, and final cup placement was performed based on intraoperative feedback on inclination and anteversion angles according to the preoperative planning [9].

### CT-based navigation THA

The CT-based navigation system (CT-Hip System V1.1, Stryker) guided cup placement. Three 4.0 mm tracker pins were inserted into the same-side iliac crest, and surface mapping included the inner acetabular wall. Reaming was performed 1 mm smaller than the preoperative plan, with manual adjustments made to ensure accurate placement, guided by real-time feedback on inclination, anteversion, and center of rotation.

### Portable navigation THA

The AR Hip (Zimmer Biomet) used a smartphone-based navigation tool to project cup placement angles in real-time on the display. Two 3.2 mm tracker pins were placed in the same-side iliac crest, and two anatomical landmarks (bilateral anterior superior iliac spines) were registered. The smartphone’s gyro sensor automatically detected the gravitational vector, providing real-time inclination and anteversion angles for manual component insertion [22].

### CT-based analysis

All patients underwent postoperative hip joint CT scans (from the anterior superior iliac spine to the knee joint through the distal femoral condyles) using a 320-row multi-detector helical CT scanner (detector Beam collimation, 80 × 0.5, 40 mm; slice thickness, 1.00 mm; slice interval, 1.00 mm; Aquilion ONE, Toshiba Medical healthcare, Tochigi, Japan) at 3 months postoperatively [23].

**Fig. 1:**
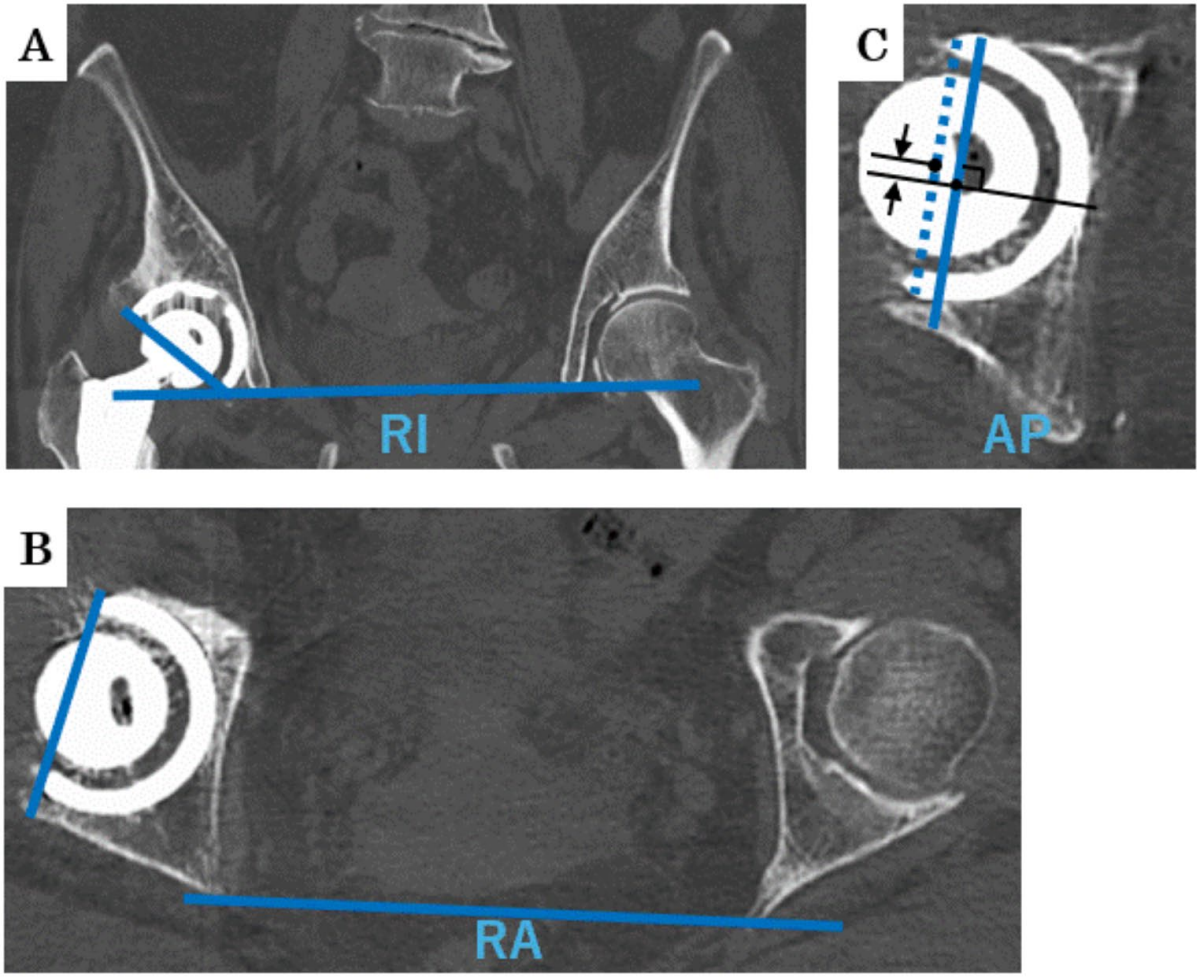
Postoperative CT scans were used to measure cup placement angles and positions. (**A**) The coronal view illustrates the measurement of radial inclination (RI), indicating cup tilt relative to the vertical axis. (**B**) The axial view demonstrates the measurement of radial anteversion (RA), indicating the forward tilt of the cup. (**C**) The axial view shows the evaluation of the anterior-posterior cup position (AP), assessed by measuring the absolute distance between the center of the acetabulum and the center of the cup

Postoperative CT scans were used to measure cup placement angles (radiographic inclination [RI] and radiographic anteversion [RA]), and accuracy was evaluated by comparing the absolute differences with preoperative target angles. The Anterior-posterior (AP) cup position was evaluated by measuring the absolute distance between the acetabular and cup center position in the axial view of the postoperative CT scans (Fig. 1). Measurements were taken by two independent observers to assess intraobserver and interobserver reliabilities. The intraobserver reliabilities (intraclass correlation coefficients) were 0.943 for inclination and 0.950 for anteversion, while the interobserver reliabilities were 0.931 for inclination and 0.919 for anteversion.

### Patient-reported outcomes

Clinical evaluation was conducted using the Japanese Orthopaedic Association Hip Disease Evaluation Questionnaire (JHEQ), a PROM for assessing the clinical condition of patients with hip disorders. The total score ranges from 0 (worst) to 84 points (best). The JHEQ includes three subscales—namely, pain (0–28 points), movement (0–28 points), and mental (0–28 points)—with 0 points representing the worst outcome and 28 points representing the best outcomes for each subscale [28]. The JHEQ was administered preoperatively and at 3 and 12 months postoperatively.

### Statistical analysis

The sample size was calculated based on an effect size of f = 0.4 using a priori power analysis with a one-way ANOVA (alpha level of 0.05). A total sample size of 102 participants (34 participants per group) was required to achieve 95% statistical power for detecting significant differences among the three groups, with ΔRI and ΔRA as the primary outcomes.

Statistical analyses were performed using Statcel, the Useful Addin Forms on Excel, 4th ed. (OMS Publishing, Tokyo, Japan). Cup placement angles, placement positions, and patient backgrounds were compared among the three groups using the Kruskal–Wallis test. Subsequently, for significant differences, the Dunn–Bonferroni test was applied. Categorical variables were compared using the chi-squared test. Results are presented as mean ± standard deviation (range). For all analyses, statistical significance was set at *P* value < 0.05.

## Results

Patient demographics and postoperative clinical outcomes are summarized in Table 1. The Mako group included 73 patients (73 hip joints; 6 men, 67 women; mean age: 70.1 years) with hip OA in 58 hips and ONFH in 15 hips. The CT-navi group included 83 patients (83 hip joints; 37 men, 46 women; mean age: 65.4 years) with hip OA in 69 hips and ONFH in 14 hips. The AR-navi group had 40 patients (46 hip joints; 7 men, 33 women; mean age: 67.1 years) with hip OA in 34 hips and ONFH in 6 hips. Although the three groups showed differences in follow-up time owing to differences in the period of device use (*P* = 0.009), there were no significant differences in age, sex, primary diagnosis, or Body Mass Index across the groups. Additionally, there were no significant differences in surgical time or blood loss among the groups. No postoperative complications, including dislocation, infection, or fracture, were observed in any group. Additionally, no readmissions or revisions were required in any of the groups (Table 1).

**Table 1:** Characteristics of patients and clinical outcomes

		Mako group	CT-navi group	AR-navi group	*P*-value
Patient	(hips)	73	83	46	
Age	(years)	70.0 ± 7.4 (45 to 84)	65.4 ± 10.1 (44 to 85)	67.1 ± 9.1 (53 to 85)	0.075
Sex	(Men/Women) (n)	6/67	37/46	8/38	0.093
Primary disease	(OA/ON) (n)	58/15	69/14	40/6	0.823
BMI	(kg/m^2^)	25.3 ± 4.6 (21.3 to 33.2)	23.9 ± 4.9 (18.2 to 45.2)	24.9 ± 4.9 (15.7 to 31.2)	0.351
Surgical time	(min)	78.0 ± 17.5 (52 to 120)	81.5 ± 11.9 (53 to 109)	79.5 ± 18.2 (53 to 118)	0.060
Blood loss	(mL)	255.6 ± 110.5 (70 to 550)	286.5 ± 103.4 (120 to 500)	274.7 ± 111.9 (100 to 500)	0.233
Follow-up period	(days)	571.1 ± 145.8 (377 to 790)	626.5 ± 151.4 (378 to 846)	448.9 ± 44.9 (370 to 525)	0.009
Postoperative Complications					
Dislocation	(n)	0	0	0	
Infection	(n)	0	0	0	
Fracture	(n)	0	0	0	
Readmission	(n)	0	0	0	
Revision	(n)	0	0	0	

Data are shown as mean ± standard deviation (range). Mako, robotic-assisted system; CT-navi, computed tomography-based navigation; AR-navi, augmented reality-based navigation; BMI, Body Mass Index

The cup placement angles are summarized in Table 2. The target angles for cup placement were similar across the groups, with no significant differences observed. The target RI was 40.1° in the Mako group, 40.0° in the CT-navi group, and 40.1° in the AR-navi group, while the target RA was 15.1°, 15.1°, and 14.9°, respectively. The postoperative RI was 40.4° in the Mako group, 39.6° in the CT-navi group, and 41.1° in the AR-navi group. The postoperative RA was 16.6°, 16.9°, and 17.4°, respectively. No significant differences were observed among the three groups for these angles (Table 2).

**Table 2:** Acetabular cup placement angles of each group

		Mako group	CT-navi group	AR-navi group	*P*-value
Target angle					
RI	(°)	40.1 ± 0.6 (40 to 45)	40.0 ± 0.7 (38 to 45)	40.1 ± 0.7 (40 to 45)	0.773
RA	(°)	15.1 ± 0.9 (10 to 20)	15.1 ± 1.3 (8 to 19)	14.9 ± 0.4 (12 to 15)	0.340
Postoperative					
RI	(°)	40.4 ± 1.3 (38 to 44)	39.6 ± 2.5 (32 to 47)	41.1 ± 2.7 (36 to 46)	0.252
RA	(°)	16.6 ± 1.7 (12 to 21)	16.7 ± 2.6 (8 to 23)	17.4 ± 3.4 (11 to 26)	0.392

Data are shown as mean ± standard deviation (range). RI, Radiographic Inclination; RA, Radiographic Anteversion; Mako, robotic-assisted system; CT-navi, computed tomography-based navigation; AR-navi, augmented reality-based navigation

Cup placement accuracy for both angle and position is shown in Table 3. Significant differences were observed in ΔRI (Mako group: 0.7°, CT-navi group: 1.6°, AR-navi group: 2.2°) and ΔRA (Mako group: 1.8°, CT-navi group: 2.4°, AR-navi group: 3.3°) among the groups (*P* < 0.001, *P* = 0.004). The Dunn–Bonferroni test revealed that ΔRI in the Mako group was significantly lower compared to both the CT-navi (*P* = 0.002) and AR-navi groups (*P* < 0.001), with no significant difference between the CT-navi and AR-navi groups. For ΔRA, the Mako group showed significantly smaller differences compared with the AR-navi group (*P* = 0.003), with no differences between the CT-navi group and the other groups. In addition, significant differences were observed in the ΔAP cup position among the groups, with values of 0.9 mm in the Mako group, 1.3 mm in the CT-navi group, and 2.6 mm in the AR-navi group (*P* < 0.001). The Dunn–Bonferroni test revealed that the Mako (*P* < 0.001) and CT-navi group (*P* = 0.021) had significantly smaller position differences compared to the AR-navi group (Table 3). Post hoc power analysis using the observed effect sizes (f = 0.344 for ΔRI and f = 0.296 for ΔRA) confirmed statistical power of 0.9945 and 0.9702, respectively, indicating that the study had sufficient power to detect significant between-group differences for these parameters.

**Table 3:** Acetabular cup placement angles of each group

		Mako group	CT-navi group	AR-navi group	*P*-value
ΔRI	(°)	0.7 ± 1.1 (0 to 4.0)	1.6 ± 1.7^*^ (0 to 6.0)	2.2 ± 1.8^**^ (0 to 6.0)	< 0.001
ΔRA	(°)	1.8 ± 1.5 (0 to 5.0)	2.4 ± 1.9 (0 to 8.0)	3.3 ± 2.6^**^ (0 to 11.0)	0.004
ΔAP	(°)	0.7 ± 0.5 (0.1 to 2.0)	1.1 ± 0.6 (0.4 to 3.1)	1.9 ± 1.0^**, #^ (0.59 to 4.2)	< 0.001

Data are shown as mean ± standard deviation (range). ΔRI, The absolute difference between the post-operative Radiographic Inclination and the preoperative target angle; ΔRA, The absolute difference between the post-operative Radiographic Anteversion and the preoperative target angle; ΔAP, absolute distance of anterior and posterior cup positions; Mako, robotic-assisted system; CT-navi, computed tomography-based navigation; AR-navi, augmented reality-based navigation *, vs. Mako group *P* < 0.05; **, vs. Mako group *P* < 0.01; #, vs. CT-navi group *P* < 0.05

The JHEQ scores, including the total score and subscales for Pain, Movement, and Mental, were recorded preoperatively and at 3 and 12 months postoperatively (Table 4). No significant differences were found in the preoperative JHEQ total score or the Pain, Movement, or Mental subscales among the groups. Similarly, no significant differences were observed in these scores at 3 and 12 months postoperatively across the groups (Table 4).

**Table 4:** Comparison of JHEQ scores among the groups

Time Point	Subscale		Mako group	CT-navi group	AR-navi group	*P*-value
Preoperatively	Total score	(pts)	22.1 ± 11.8 (0 to 57)	23.3 ± 14.0 (1 to 60)	18.0 ± 9.5 (1 to 44)	0.132
	Pain	(pts)	7.5 ± 4.7 (0 to 20)	7.4 ± 6.3 (0 to 22)	6.6 ± 4.5 (0 to 22)	0.433
	Movement	(pts)	5.1 ± 4.5 (0 to 18)	6.0 ± 5.1 (0 to 21)	4.3 ± 3.4 (0 to 13)	0.449
	Mental	(pts)	9.0 ± 5.3 (0 to 26)	9.4 ± 5.9 (0 to 26)	7.6 ± 5.1 (0 to 19)	0.352
Postoperatively	Total score	(pts)	61.0 ± 16.0 (27 to 84)	63.1 ± 14.0 (33 to 84)	61.7 ± 14.0 (33 to 84)	0.871
3 months	Pain	(pts)	23.4 ± 4.5 (13 to 28)	23.7 ± 5.2 (9 to 23)	23.3 ± 5.1 (14 to 28)	0.577
	Movement	(pts)	16.0 ± 6.7 (3 to 28)	16.8 ± 6.7 (3 to 28)	15.7 ± 6.8 (4 to 28)	0.715
	Mental	(pts)	21.4 ± 7.3 (3 to 28)	22.0 ± 5.6 (7 to 28)	21.8 ± 5.4 (11 to 28)	0.891
Postoperatively	Total score	(pts)	64.6 ± 11.4 (50 to 78)	63.8 ± 13.5 (46 to 84)	70.0 ± 11.0 (47 to 84)	0.341
12 months	Pain	(pts)	26.3 ± 2.8 (21 to 28)	26.4 ± 3.4 (20 to 28)	26.5 ± 2.3 (22 to 28)	0.863
	Movement	(pts)	16.4 ± 4.9 (10 to 23)	18.7 ± 7.3 (8 to 28)	18.8 ± 6.6 (4 to 28)	0.463
	Mental	(pts)	21.6 ± 5.6 (14 to 28)	22.1 ± 5.5 (11 to 28)	23.1 ± 3.9 (16 to 27)	0.933

Data are shown as mean ± standard deviation (range). JHEQ, Japanese Orthopaedic Association Hip Disease Evaluation Questionnaire; Mako, robotic-assisted system; CT-navi, computed tomography-based navigation; AR-navi, augmented reality-based navigation; pts, points

## Discussion

To our knowledge, this is the first study to compare the accuracy of acetabular cup placement using three different CAE systems—robotic-assisted system, CT-based navigation, and portable navigation—in THA under identical postoperative rehabilitation protocols. Our study is novel in evaluating both the precision of these systems and their impact on early PROMs. We found that the robotic-assisted system provided the highest accuracy in acetabular cup placement, significantly reducing deviations in both RI and RA compared to the other systems. Despite these differences in accuracy, there was no significant variation in PROMs between the groups at 12 months postoperatively. This suggests that early postoperative PROMs may not be directly influenced by the precision of cup placement.

Previous studies have highlighted the utility of CAE in THA, particularly in achieving accurate cup placement angles through comparative analysis of different systems. For instance, Tsutsui et al. demonstrated that a CT-based navigation system provided more precise cup placement than conventional mechanical guides in THA [29]. Furthermore, Sato et al. and Hayashi et al. reported average absolute differences for cup placement using the robotic-assisted system at 1.1° ± 1.0° and 1.8° ± 2.0° for inclination, and 1.2° ± 1.1° and 1.9° ± 2.3° for anteversion [10, 30]. Ando et al. compared the robotic-assisted system with CT-based navigation, noting that the robotic-assisted system offered greater accuracy in cup placement angles [7]. Notably, screw fixation, which can affect cup orientation, was avoided in the robotic-assisted group, contributing to less variability in cup angle compared to the CT-based navigation group [31]. The benefits of portable navigation have also been highlighted in the literature. Ogawa et al. reported installation accuracy of 1.9° for RI and 2.8° for RA using AR navigation, both demonstrating improved accuracy over conventional methods. Similarly, Shimizu et al. found that cup placement accuracy using AR navigation was 2.6 ± 2.1° for RI and 3.9 ± 3.0° for RA, demonstrating a significant reduction in RI installation error compared to traditional techniques [32]. While previous studies have compared two systems, no study has yet compared all three systems simultaneously.

In our study, the robotic-assisted system demonstrated the highest cup placement accuracy compared to both the CT-based and portable navigation systems, with significantly smaller deviations in both RI and RA. These findings further emphasize the potential of advanced CAE systems in enhancing surgical precision in THA.

Several studies have examined cup positioning accuracy in THA using CAE. Tsutsui et al. and Iwana et al. demonstrated that CT-based navigation provided significantly higher spatial precision than conventional methods. The mean discrepancies were approximately 1.9 mm in the mediolateral, AP, and superoinferior (SI) positions [29, 33]. Furthermore, Ando et al. found that the absolute error of the center of rotation in the AP and SI directions was significantly smaller in the robotic-assisted THA than in the CT-based navigation [7].

One contributing factor is that CT-based navigation displays the target position during reaming, allowing surgeons to ream while observing the screen. In the robotic-assisted system, a robotic arm ensures precise reaming to the target position with a single-size reamer, reducing residual bone in the reaming pathway. The robotic arm also stabilizes the cup impactor during insertion, improving cup positioning accuracy. In contrast, portable navigation systems lack positional tracking during reaming and rely on manual techniques, increasing the risk of positional deviations. Insufficient acetabulum rim reaming and the use of progressively larger reamers can leave residual bone, leading to cup displacement. Previous studies have indicated that angular changes during manual press-fit cup insertion significantly cause inaccurate positioning, and the stress balance between the cup and surrounding bone can further influence these changes [34].

Our study confirmed that both the robotic-assisted system and CT-based navigation systems demonstrated smaller positional deviations in cup placement compared with portable navigation. Notably, the robotic-assisted system had the smallest errors in the AP position, as determined by postoperative CT scans. Although cup positioning in the AP direction is less frequently discussed compared with inclination and anteversion, its importance cannot be underestimated. Accurate cup positioning in the AP direction is essential for optimizing the press-fit fixation between the cup and the acetabulum, which enhances the initial stability of the implant [35]. This stability is crucial for long-term osseointegration, reducing the risk of aseptic loosening, and preventing complications such as dislocation or wear over time [36]. Moreover, while the AP position is often considered in terms of maximizing bone coverage, studies have shown that it also plays a significant role in the mechanical stability of the hip joint. Precise AP positioning is linked to reducing the risk of iliopsoas impingement and bony impingement, which can result in persistent groin pain, limited hip function, and lower patient satisfaction [37]. Excessive anterior or posterior deviations in cup placement are associated with soft tissue irritation and impingement, further underscoring the need for accuracy in this direction [38, 39]. In fact, robotic-assisted systems have been shown to improve cup placement precision by stabilizing both the reaming and cup insertion processes, thus minimizing positional deviations and their associated risks [40]. By ensuring accurate AP positioning, these systems contribute to reducing the incidence of complications and enhancing the long-term stability of the artificial hip joint.

Several systematic reviews and meta-analyses, including those by Kort et al. and Samuel et al., have consistently reported no statistically significant differences in functional outcomes or clinical scores between conventional and robotic-assisted THA [41, 42]. Specifically, Han et al. examined weighted Harris Hip Score (HHS), Postel-Merle d’Aubigné (PMA) scores, and pooled outcomes, such as the Japanese Orthopedic Association score, without identifying significant differences [43]. Similarly, Karunaratne et al. and Chen et al. reported comparable findings when analyzing pooled PMA, HHS, and modified HHS scores [44, 45]. Singh et al. also compared PROMs across robotic-assisted systems, CT-based navigation, and conventional methods, concluding that the differences between these modalities were not clinically significant in early patient-reported outcomes [24].

Consistent with previous studies, our study demonstrated no significant differences in PROMs between the three CAE systems, as measured by JHEQ at 12 months postoperatively. Shoji et al. found that implant-related factors affecting postoperative PROMs in THA, such as combined anteversion and stem anteversion, were associated with the JHEQ total score and Movement subscale [23]. This suggests that short-term PROMs may not be solely influenced by differences in cup placement accuracy among CAE systems. Our study supports this view, as we found no significant differences in PROMs between the CAE systems, despite variations in cup placement accuracy.

However, it is essential to recognize that while early postoperative outcomes might not fully reflect the impact of surgical precision, long-term cup placement accuracy could affect joint function and the risk of revision surgery [46]. Ong et al. demonstrated that differences in PROMs can be observed depending on the type of robotic system used [47], and Peters et al. reported that differences in surgical approach, such as the direct anterior approach versus the posterolateral approach, have the potential to affect short-term PROMs [48]. Therefore, long-term follow-up studies, including the influence of surgical approach and robotic system design, are crucial to understand these relationships. In addition, PROMs should account for factors that might influence patient satisfaction, such as postoperative rehabilitation and patient expectations [49, 50]. These factors may play a significant role in determining overall satisfaction, independent of the surgical technique used [51].

This study has certain limitations. First, as a single-center, retrospective study, the external validity of the findings may be limited, and there is a potential risk of selection bias. Second, although a priori power analysis was performed to confirm that the sample size was sufficient for detecting significant differences, the relatively small overall sample size, particularly in the AR-navi group, may still have influenced the statistical power for some comparisons. However, when compared to previous reports on portable navigation, the deviation in cup placement angles was smaller, suggesting minimal impact from the sample size [32]. Third, the relatively short follow-up period of 12 months limits our ability to draw conclusions about long-term outcomes, including complications. Additionally, all procedures were performed by an experienced surgeon, which may have influenced the results, as previous studies have reported differences in cup placement accuracy between residents and supervising surgeons [52]. Therefore, the use of CAE by less experienced surgeons could potentially impact PROMs. Finally, the lack of detailed intraoperative data limits the ability to assess small procedural variations that may have affected outcomes.

In conclusion, THA with the robotic-assisted system demonstrated superior accuracy in acetabular cup placement compared to CT-based and portable navigation systems. Although no significant differences were observed in short-term PROMs, accurate cup placement may reduce complications and improve long-term outcomes. Future multicenter, randomized controlled trials are needed to further validate our findings and assess the potential long-term effects of robotic assistance in THA. Overall, our study highlights the value of integrating innovative technologies into THA procedures to enhance surgical precision and patient satisfaction, ultimately improving patient outcomes and quality of life for patients.

## Data Availability

No datasets were generated or analysed during the current study.
